# Neutrophil-to-Lymphocyte Ratio Dynamics From Pre-diagnosis to End-Stage Amyotrophic Lateral Sclerosis (ALS): A Case Study on Association With Progression and Clinical Events

**DOI:** 10.7759/cureus.58109

**Published:** 2024-04-12

**Authors:** Kevin De Jesus-Morales, Wilfredo De Jesús-Rojas, Marcos J Ramos-Benitez

**Affiliations:** 1 Department of Physical Medicine, Rehabilitation, and Sports Medicine, University of Puerto Rico – Medical Sciences Campus, San Juan, PRI; 2 Department of Basic Sciences, Ponce Health Sciences University, Ponce, PRI

**Keywords:** inflammation, lymphocyte, neutrophil, amyotrophic lateral sclerosis, als, nlr

## Abstract

Amyotrophic lateral sclerosis (ALS) is a neurodegenerative condition characterized by the progressive degeneration of motor neurons, resulting in muscle weakness and paralysis. The neutrophil-to-lymphocyte ratio (NLR) has emerged as a potential marker for monitoring disease severity and progression in ALS, yet longitudinal analyses of NLR are limited. Our study conducts an in-depth examination of NLR dynamics from before diagnosis through the disease's progression to its end stage. We analyze the case of a 56-year-old Puerto Rican male with ALS, tracking his NLR over 13 years - six years before and seven years after his diagnosis - alongside assessments of clinical symptoms and lung function. Our findings indicate that NLR values were initially normal but significantly increased with the onset of symptoms. NLR remained elevated above the normal range, with a notable exception during a period of edaravone therapy when levels normalized. The study demonstrates a clear elevation in NLR associated with ALS progression and critical clinical events, such as symptom onset, diagnosis, and the initiation of respiratory support. This research is, to our knowledge, the first to provide a detailed characterization of NLR changes from the pre-diagnostic phase to end-stage ALS, showing its correlation with clinical deterioration, decreased pulmonary function, and key clinical events. Our results contribute to the body of evidence on NLR's role in ALS while enhancing our understanding of ALS's natural progression.

## Introduction

Amyotrophic lateral sclerosis (ALS), often referred to as Lou Gehrig's disease, is a neurodegenerative disorder characterized by the progressive degeneration of motor neurons in the brain and spinal cord. This leads to muscle weakness, atrophy, and ultimately, death, usually due to acute-on-chronic respiratory failure within two to four years of diagnosis, but 10-20% of ALS patients have a survival longer than 10 years [1]. The pathogenesis of ALS is complex, involving disruptions in RNA processing, protein aggregation, oxidative stress, and mitochondrial dysfunction [2]. Although the etiology of ALS remains poorly understood, immune responses, particularly inflammation, are increasingly recognized as contributing factors to disease progression [3,4]. Beyond the nervous system, accumulating evidence suggests that ALS is associated with systemic immune dysregulation and peripheral inflammation. Alterations in peripheral immune cell populations, cytokine profiles, and immune signaling pathways have been observed in ALS patients and animal models, indicating a broader involvement of the immune system in disease pathogenesis [5]. This involvement could have dual implications, serving both as a contributor to disease progression and as a means to monitor the disease.

One of the primary objectives at the time of diagnosis is to identify prognostic factors at the earliest stage feasible, providing patients with insights and enabling clinicians to plan to start the appropriate interventions. The neutrophil-to-lymphocyte ratio (NLR) is a hematological parameter calculated from the ratio of the neutrophil count to the lymphocyte count, serving as a marker for systemic inflammation. The NLR is calculated using the following formula, NLR = (Absolute neutrophil count, cells/μL)/(Absolute lymphocyte count, cells/μL) (Note: neutrophil and lymphocyte values can also be entered as percentages). Elevated NLR values often indicate a predominance of pro-inflammatory processes. Normal NLR values typically fall within the range of 1 to 2 while values above 3 or below 0.7 in adults are considered pathological. NLR values between 2.3 and 3.0 may indicate a gray zone, potentially serving as an early warning sign of underlying pathological conditions such as cancer, atherosclerosis, infection, or inflammation [6].

In ALS, the NLR has emerged as a potential prognostic biomarker, correlating to disease progression, severity, and survival rates [7,8]. Despite the importance of NLR in ALS, longitudinal studies following its dynamics are scarce. To our knowledge, no study has longitudinally documented NLR prior to symptoms onset and through disease progression until end-stage, which is crucial to establish temporal relationships between leukocyte dynamics (e.g. neutrophils) and the expected clinical declining timeline. To address this, we present a case report that provides a 13-year retrospective longitudinal assessment of NLR in a sporadic ALS patient.

## Case presentation

A 56-year-old Puerto Rican male, without a past medical history, was in his usual state of health consisting of independent activities of daily living (ADLs) and free ambulation until 2015, when he started experiencing intermittent episodes of severe cramps in both legs during the night. Within six months, he started dragging his right foot due to progressive leg weakness (right > left). Neurological tests revealed significant bilateral lower extremity weakness, lesser in upper extremities, and fasciculations in all limbs and paraspinal muscles. Sensory functions were intact, with no bulbar symptoms. He showed symmetrical hyperreflexia and ankle clonus. Mobility drastically declined, and he needed assistance to stand and the use of a 1-point cane, rollator, and wheelchair for movement.

As part of the diagnostic workup, the patient underwent imaging of the brain and cervical and thoracic spine MRI with unremarkable results. An array of laboratory investigations, including heavy metals, autoimmune evaluation, and HIV serology were all normal. Creatine phosphokinase (CPK) and aldolase (8.30 U/L) levels were increased (941 U/L) one month prior to diagnosis. Three electrodiagnostic studies (nerve conduction study and electromyography) were performed with inconsistent results, but the last study showed findings consistent with motor neuron disease, showing fasciculations in all spinal levels. Approximately 18 months after symptom onset (2017), he was diagnosed with ALS. Genetic testing at the time of diagnosis was not performed as upper motor neuron symptoms were evident.

**Figure 1:**
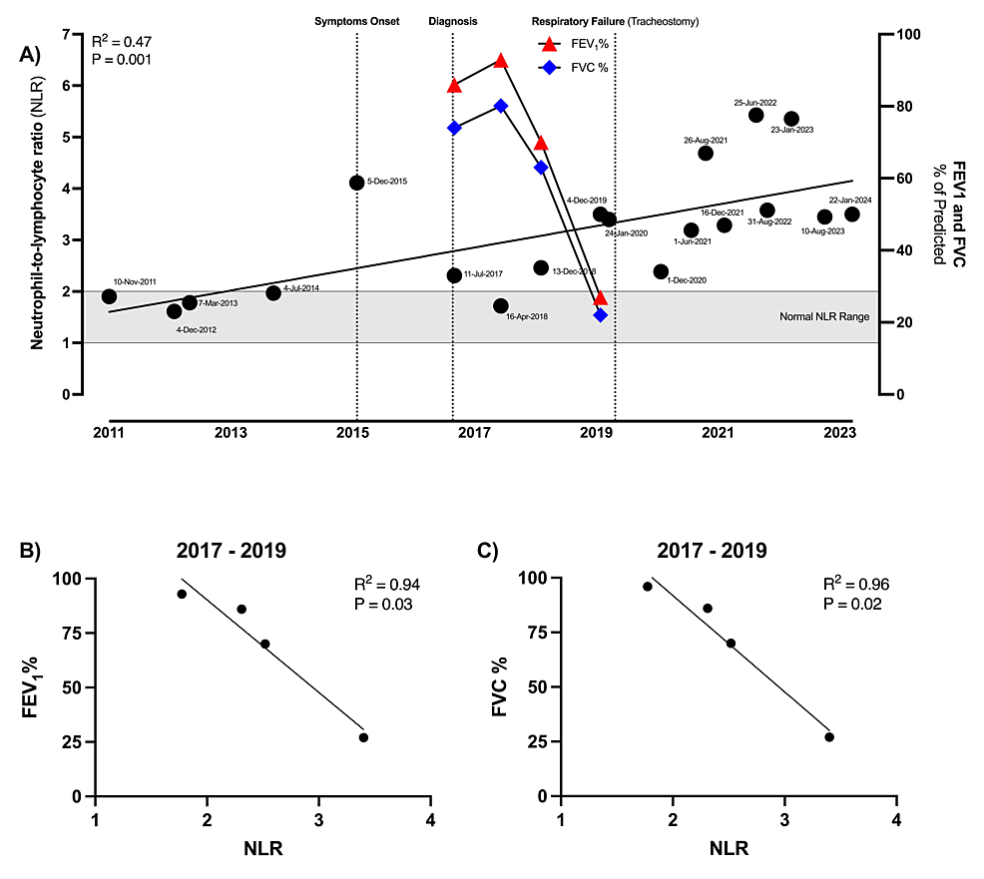
Association between NLR, disease progression, and decline in pulmonary function A) This scatter plot depicts the neutrophil-to-lymphocyte ratio (NLR) of the amyotrophic lateral sclerosis (ALS) patient over a period from November 2011 to January 2024 and forced expiratory volume in 1 second (FEV1) and forced vital capacity (FVC) (% of predicted) values from July 2017 to December 2019. Each black dot represents the NLR value at a specific date. Linear regression analysis showed an upward trend in NLR over the 13-year period (R^2^=0.47, P=0.001, 95% CI). FEV1 and FVC (% of predicted) values are shown in red and blue, respectively. Key clinical events are annotated as dotted lines. The normal NLR range is shown as a gray area. B and C) Pearson correlation coefficient was used to assess the linear relationship between NLR and FEV1 or FVC, with a 95% confidence interval.

The pulmonary function test (PFT) showed a maximal inspiratory pressure (MIP) of 31 (predicted value 90.0 cmH_2_O) and maximal expiratory pressure (MEP) of 55 (predicted value 134.7 cmH_2_O) consistent with severe neuromuscular weakness. Following the diagnosis of ALS, the initiation of pulmonary function monitoring was undertaken to assess the patient’s respiratory capacity. From 2 to 26 months post-diagnosis (2017-2019), longitudinal measurements of forced expiratory volume in 1 second (FEV1) and forced vital capacity (FVC) revealed a significant decline. FEV1 decreased from 86% to 27% of the predicted values while FVC dropped from 74% to 22% of the predicted values, indicating a substantial reduction in respiratory capacity (Figure 1A, red and blue line, respectively). This trend of decreasing FEV1 and FVC over time was consistent with functional deterioration. Around 18 months post-diagnosis (2019), the patient was started on non-invasive positive pressure ventilation (BiPAP) based on PFT results. At 34 months post-diagnosis (2020), the patient had an acute episode of altered mental status and respiratory distress that led to emergency intubation and mechanical ventilation due to hypercapnic respiratory failure, which ended in the need for a tracheostomy and percutaneous endoscopic gastrostomy (PEG) placement. Since then, the patient has been in bed confinement status and in need of maximal assistance for ADLs.

A retrospective analysis of the patient’s NLR was conducted, utilizing complete blood count (CBC) data from November 2011 (six years pre-diagnosis) until January 2024 (seven years post-diagnosis). We correlated that data with PFTs, as an indicator of disease progression, and key clinical events. All the CBC analyses conducted for this study had white blood cell counts within the normal range, limiting the possibility of underlying infections influencing NLR. The analysis revealed a significant upward trend in the NLR as the disease progresses (R^2^=0.47, P=0.001) (Figure 1A). This longitudinal analysis of NLR values also revealed a notable surge in NLR to levels (>4) at the time of symptom onset. After this surge, levels remained elevated, exceeding the normal range of 1 to 2 [6]. During the 10-12 months post-diagnosis (2018), we observed a transient decline in NLR to normal levels and a slight increase in FEV1 and FVC. These changes align with the duration of treatment with edaravone. During the period of most rapid disease progression (time of diagnosis to 30 months post-diagnosis), the NLR negatively correlated with the percent predicted value of FEV1 (R^2^=0.95, P=0.02) and FVC (R^2^=0.96, P=0.01) (Figures 1B-1C). During the late to end-stage of the disease, NLR levels stayed above the normal range, with fluctuations between 2.4 and 5.4.

## Discussion

To our knowledge, this study represents the first documentation of longitudinal changes in NLR in correlation with ALS progression that incorporates data from pre-diagnosis to end-stage. Initial observations in our case revealed NLR within normal limits before symptom onset, with a significant sharp increase coinciding with the initial clinical manifestations in the form of severe leg cramps. Interestingly, a recent study evaluating a total of 345,000 individuals showed that higher levels of neutrophils and NLR were linked to an increased risk of developing ALS [9], proposing that changes in NLR can be detected in the absence of evident motor neuron deficits. Also, increased NLR was associated with a more rapid disease course, shorter survival time, and elevated mortality rates in ALS patients [8,10,11]. Our study enhances current understanding by monitoring a patient's disease progression, revealing that NLR may increase in the early stages of ALS and subsequently correlate with clinical deterioration. Accordingly, NLR has also been described as an indicator of inflammation in other neurodegenerative conditions like Parkinson's disease [12]. In multiple system atrophy, elevated NLR levels correlate with central neurodegeneration, increased clinical burden, and shorter survival durations [13]. Patients with early multiple sclerosis exhibited elevated NLR levels compared to healthy controls, with NLR showing a weak correlation with the multiple sclerosis severity score [14]. This observation raises the fundamental question of whether elevated NLR reflects a driver of ALS pathogenesis via neutrophil-induced damage or a byproduct of underlying inflammatory activity.

Following the initial increase, the NLR showed a short period of normalization. This temporary reduction in NLR coincides with slight improvements in pulmonary function, suggesting a possible inflammatory modulation in response to therapeutic intervention with edaravone. Such dynamic shifts in NLR levels in response to treatment have been documented in other pathologies, where changes in NLR were reflective of treatment responses in cancer [15,16]. In ALS, our case is the first report to hint at the dynamic nature of NLR in relation to therapeutic interventions, a phenomenon that warrants further investigation to elucidate the role of NLR in informing disease management.

While the link between NLR and ALS progression has been previously documented, our case study offers distinctive insights by comprehensively documenting NLR dynamics from several years before diagnosis until the end stage. This type of detailed extended timeline offers a valuable perspective on how NLR could change in response to clinical events and treatments while demonstrating its consistent inverse relationship with disease progression and pulmonary function decline. Such findings prompt a reevaluation of NLR's role beyond a prognostic biomarker, potentially serving as a real-time indicator of disease activity and response to treatment.

Lastly, elevated NLR in ALS has been linked to dysregulated inflammatory pathways, marked by increased pro-inflammatory cytokines and neutrophil infiltration into the central nervous system (CNS), which are thought to exacerbate neurodegeneration [17]. The relationship between heightened NLR and ALS progression is further complicated by the observation that systemic inflammation, particularly through interleukin 6-mediated mechanisms, may contribute to the breakdown of the CNS barrier [18]. In ALS and other diseases, neutrophils play a detrimental role in skeletal muscle, where their infiltration and interaction with mast cells lead to neuromuscular junction denervation and myofiber atrophy, processes that are exacerbated by neutrophil-derived proteases and reactive oxygen species, perhaps contributing to the disease progression and severity [19,20]. Furthermore, lymphocytes play a multifaceted role in ALS, with regulatory T cells (Tregs) generally providing neuroprotection, which is compromised in ALS patients [21]. Conversely, cytotoxic CD8+ T cells may contribute to the death of motor neurons [22]. Additionally, animal models have shown that the early presence of lymphocytes may be harmful, while at later stages, they may be neuroprotective [23], indicating the complexity and stage-dependence of lymphocyte roles in ALS pathology. These insights not only reinforce the significance of NLR as a potential biomarker for ALS but also highlight the potential role of targeted therapeutic strategies aimed at mitigating inflammation-driven neurodegeneration in ALS, offering a promising avenue for delaying ALS progression trajectory and improving patient outcomes.

## Conclusions

This case study further expands on the previously reported potential of NLR as an early marker for disease progression in ALS. Our results show that an increase in NLR not only precedes more apparent clinical symptoms but also correlates with disease severity and responds to therapeutic interventions. Thus, the study provides a rationale to further evaluate NLR as a tool for monitoring disease in its early stages, its progression, and the effectiveness of treatments. A limitation of this study is its reliance on a single retrospective case, which may restrict available data and can also lead to recall bias from key clinical events. This may limit the generalizability of the findings to a broader ALS population. However, this report enhances our understanding of ALS pathophysiology and provides further rationale for studies. Future longitudinal cohort studies are necessary to validate our observations from this case study and to further investigate the potential of NLR in the early stages of the disease and its role in informing therapeutic decisions, with the aim of improving outcomes for ALS patients.
